# Prognostic value of D-dimer for adverse outcomes in patients with infective endocarditis: an observational study

**DOI:** 10.1186/s12872-021-02078-3

**Published:** 2021-06-05

**Authors:** Ying-Wen Lin, Mei Jiang, Xue-biao Wei, Jie-leng Huang, Zedazhong Su, Yu Wang, Ji-yan Chen, Dan-qing Yu

**Affiliations:** 1 Department of Cardiology, Guangdong Cardiovascular Institute, Guangdong Provincial Key Laboratory of Coronary Heart Disease Prevention, Guangdong Provincial People’s Hospital, Guangdong Academy of Medical Sciences, Guangzhou, 510080 China; 2 Shantou University Medical College, Shantou, China; 3 Department of Critical Care Medicine, Guangdong Provincial Geriatrics Institute, Guangdong Provincial People’s Hospital, Guangdong Academy of Medical Sciences, Guangzhou, 510080 China

**Keywords:** D-dimer, Infective endocarditis, Embolism, Stroke, Prognosis

## Abstract

**Background:**

Increased D-dimer levels have been shown to correlate with adverse outcomes in various clinical conditions. However, few studies with a large sample size have been performed thus far to evaluate the prognostic value of D-dimer in patients with infective endocarditis (IE).

**Methods:**

613 patients with IE were included in the study and categorized into two groups according to the cut-off of D-dimer determined by receiver operating characteristic (ROC) curve analysis for in-hospital death: > 3.5 mg/L (n = 89) and ≤ 3.5 mg/L (n = 524). Multivariable regression analysis was used to determine the association of D-dimer with in-hospital adverse events and six-month death.

**Results:**

In-hospital death (22.5% vs. 7.3%), embolism (33.7% vs 18.2%), and stroke (29.2% vs 15.8%) were significantly higher in patients with D-dimer > 3.5 mg/L than in those with D-dimer ≤ 3.5 mg/L. Multivariable analysis showed that D-dimer was an independent risk factor for in-hospital adverse events (odds ratio = 1.11, 95% CI 1.03–1.19, *P* = 0.005). In addition, the Kaplan–Meier curve showed that the cumulative 6-month mortality was significantly higher in patients with D-dimer > 3.5 mg/L than in those with D-dimer ≤ 3.5 mg/L (log-rank test = 39.19, *P* < 0.0001). Multivariable Cox regression analysis showed that D-dimer remained a significant predictor for six-month death (HR 1.11, 95% CI 1.05–1.18, *P* < 0.001).

**Conclusions:**

D-dimer is a reliable prognostic biomarker that independently associated with in-hospital adverse events and six-month mortality in patients with IE.

## Background

Despite improvements in the diagnostic approach and management strategy, infective endocarditis (IE) remains associated with high rates of in-hospital and long-term mortality, and significant complications [1–6]. Therefore, rapid identification of patients at high-risk of death could help clinical decision-making with respect to the timing of surgery and intensity of in-hospital care in order to improve prognosis.

Systemic embolization (especially stroke) is a severe complication of IE and is associated with increased morbidity and mortality [7]. Early identification of coagulation activation and thrombus formation with associated biomarkers such as D-dimer may be of prognostic value. D-dimer is a fibrin-degradation product that is released when a blood clot disintegrates, indicating thrombosis or fibrinolysis [8]. It is a valuable blood marker for the diagnosis and evaluation of a vast array of thrombosis-related clinical conditions such as venous thromboembolism, pulmonary thromboembolism, and myocardial infarction [9–11]. Turak et al. reported that increased D-dimer level at admission was associated with high in-hospital mortality in patients with IE [12]. However, the sample size of their study was small (n = 157), and the impact of D-dimer on long-term outcomes in patients with IE was not discussed. Therefore, in this study, we aimed to evaluate the association of D-dimer at admission with in-hospital and six-month outcomes in a relatively large series of IE patients.

## Method

### Study population

In this observational, 613 patients diagnosed with definite IE at Guangdong Provincial People’s Hospital between January 2009 and February 2017 were included. The diagnosis of definite IE was in accordance with the current guideline [2]. We excluded patients who were < 18 years old, lack data regarding on-admission D-dimer level, and had concomitant disseminated intravascular coagulation at admission. This study was approved by the Ethics Committee of Guangdong Provincial People’s Hospital with a waiver of written informed consent because of the retrospective study design (No. GDREC2020098H). Oral informed consent was obtained from patients or their relatives by telephone and recorded by trained nurses during the follow-up period.

### Measurement and data collection

D-dimer level was measured using quantitative latex turbidimetric test in our hospital. All subjects underwent either transthoracic (TTE) or transesophageal echocardiography (TEE) within 24 h of admission. Left ventricular ejection fraction (LVEF) was evaluated using the Simpson’s biplane method. Echocardiographic results of the type, severity of valvular involvement, perivalvular complications were documented. Estimated glomerular filtration rate (eGFR) was calculated using the four-variable Modification of Diet in Renal Disease formula for the Chinese population [13]. Surgical treatment was performed based on current guideline recommendations [2]. Patients who could not tolerate or afford the operation received conservative therapy. Patients’ clinical and demographic data including age, sex, predisposing factors, history of illness, and comorbid conditions, common laboratory results were collected from the electronic medical record by one researcher and reviewed by another researcher.

### Follow-up and outcome

All patients were followed-up by telephonic interviews at 6 months. We also reviewed the outpatient and readmission medical records of these patients. The primary outcome was in-hospital adverse events, defined as the composite of in-hospital death, stroke, and embolism. The secondary outcome was 6-month death. Stroke is diagnosed based on the presence clinical symptoms and signs of neurological deficits and radiographic evidence of ischemic or hemorrhagic changes of the brain, therefore including both ischemic and hemorrhagic stroke. Embolism was defined as the combination of ischemic stroke, pulmonary embolism or lung infarction, and arterial embolism suggested by clinical or radiographic findings.

### Statistical analysis

Statistical analyses were performed using SPSS software version 24.0 (SPSS Inc., Chicago, IL, USA). Normally distributed continuous data were expressed as mean ± standard deviation (SD) and compared using Student’s *t*-test, and non-normally distributed data were presented as median and interquartile range (IQR) and compared by the Wilcoxon rank-sum test. Categorical data were expressed as proportions and compared using the chi-squared or Fisher’s exact test. The optimal cut-off of D-dimer for predicting in-hospital death was determined by receiver operating characteristic (ROC) curve analysis. Univariable and multivariable logistic regression were used to determine risk factors for events. Variables with *P* value less than 0.05 in the univariable logistic regression analysis were included in the multivariable logistic regression analysis for in-hospital outcomes. The adjusted odds ratio (OR) and 95% confidence interval (CI) were calculated. Univariable analyses of 6-month mortality were performed using a log-rank test for patients categorized by D-dimer cutoff. Multivariable Cox regression analyses were also performed for six-month death. *P* < 0.05 was considered statistically significant for all analysis.

## Results

### Baseline clinical characteristics

In total, 613 patients (69.0% female; aged 44.9 ± 16.0 years) were included for analysis. Table 1 shows the baseline clinical characteristics of including patients. In-hospital death occurred in 58 (9.4%) patients. Patients who died during hospitalization tend to be older with a higher prevalence of diabetes mellitus (DM), rheumatic heart disease (RHD), previous valve replacement. More advanced stage of heart failure was observed in patients who expired. Dead patients had significantly higher level of CRP, SCr, D-dimer and the lower level of platelets count as compared with those who survived. Perivalvular complications under echocardiography were more common in the death group. Further, a higher proportion of patients who survived received surgical treatment than those who died.

**Table 1:** Baseline clinical characteristics in patients with or without in-hospital death

Clinical variables	All (n = 613)	Survival (n = 555)	Death (n = 58)	*P* value
*Demographics*				
Age (year), mean ± SD	44.9 ± 16.0	44.4 ± 15.8	50.0 ± 17.5	.011
Age > 60, n (%)	136 (22.2)	117 (21.1)	19 (32.8)	.042
Female, n (%)	423 (69.0)	378 (68.1)	45 (77.6)	.138
*Comorbidities*				
Hypertension, n (%)	96 (15.7)	85(15.3)	11(19.0)	.467
Diabetes mellitus, n (%)	46 (7.5)	37(6.7)	9(15.5)	.015
RHD, n (%)	109 (17.8)	93(16.8)	16(27.6)	.041
CHD, n (%)	185 (30.2)	171(30.9)	14(24.1)	.288
Prosthetic valve, n (%)	50 (8.2)	35(6.3)	15(25.9)	< .001
NYHA III-IV, n (%)	213 (34.7)	176(31.7)	37(63.8)	< .001
*Laboratory results*				
TBI (mmol/L), median (IQR)	13.7 (10.0–19.4)	13.6 (10.0–18.7)	19.8 (10.9–33.1)	< .001
CRP (mg/L), median (IQR)	34.4 (14.3–74.0)	33.0 (14.0–70.9)	66.0 (23.6–97.2)	.001
SCr (mmol/L), median (IQR)	78.0 (63.0–100.8)	77.5 (62.0–96.3)	88.9 (69.0–132.9)	.001
Platelet (10^9^/L), median (IQR)	153.0 (105.2–246.8)	158.0 (106.5–250.0)	127.9 (88.1–219.9)	.021
Fibrinogen (g/L), median (IQR)	4.7 (3.9–5.8)	4.8 (3.9–5.8)	4.3 (3.7–5.7)	.293
D-dimer^a^ (mg/L), median (IQR)	1.2 (0.5–2.5)	1.1 (0.5–2.4)	1.6 (0.8–4.0)	.002
*Echocardiographic findings*				
LVEF (%), mean ± SD	64.6 ± 7.8	64.9 ± 7.5	62.2 ± 9.7	.053
Vegetation, n (%)	592 (96.6)	541 (97.5)	51 (87.9)	< .001
Vegetation > 1 cm, n (%)	341 (68.5)	304 (67.3)	37 (80.4)	.067
Vegetation location, n (%)				.145
Aortic valve	171 (27.9)	149 (26.8)	22 (37.9)	
Mitral valve	254 (41.4)	237 (42.7)	17 (29.3)	
Others	64 (10.8)	62 (11.5)	2 (3.9)	
PVCs^b^, n (%)	64 (10.4)	50 (9.0)	14 (24.1)	< .001
Culture positive, n (%)	386 (63)	353(63.6)	33(56.9)	.314
Blood culture, n (%)	378 (61.7%)	346 (62.3)	32 (55.2)	.285
Tissue culture, n (%)	12 (2.0)	10 (1.8)	2 (3.4)	.389
Causative organisms				.455
Staphylococcus, n (%)	91 (23.4)	81 (22.9)	10 (28.6)	
Streptococcus, n (%)	206 (53.0)	191 (54.0)	15 (42.9)	
Others. n (%)	92 (23.7)	82 (23.2)	10 (28.6)	
Surgical treatment, n (%)	381 (62.2)	366(65.9)	15(25.9)	< .001

CHD, congenital heart disease; CRP, C-reactive protein; LVEF, left ventricular ejection fraction; NYHA, New York Heart Association; PVCs, perivalvular complications; RHD, rheumatic heart disease; SCr, serum creatinine; TBI, total bilirubin

^a^The normal D-dimer cut-off in the laboratory is 0.5 mg/L

^b^Perivalvular complications refer to echocardiographic findings of perivalvular leak or abscess

### D-dimer and in-hospital outcomes

**Fig. 1:**
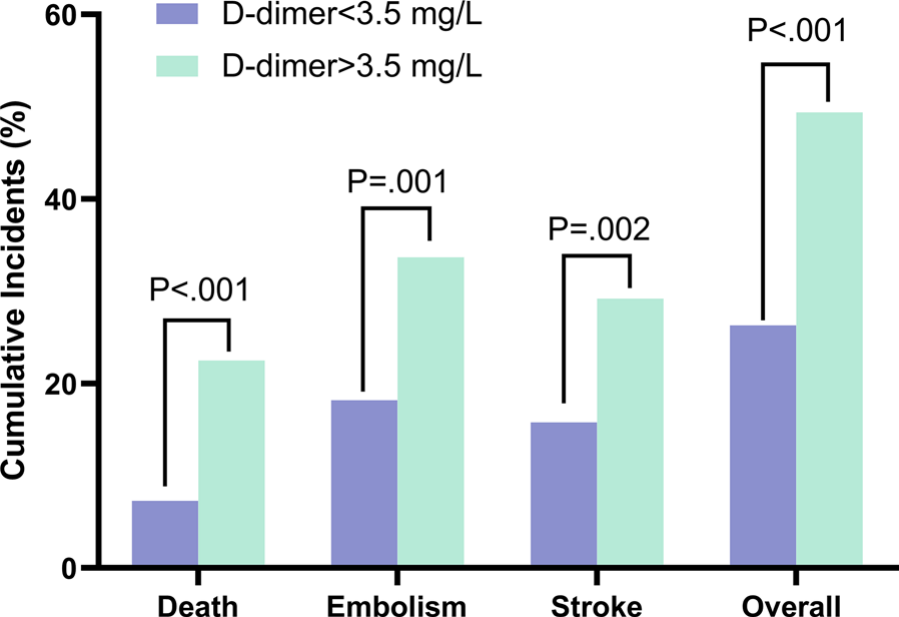
Cumulative incidences of in-hospital adverse events

The ROC curve analysis showed that D-dimer levels > 3.5 mg/L had a sensitivity of 36.2% and specificity of 87.2% (AUC = 0.622, 95% CI = 0.543–0.702, *P* = 0.002) for predicting in-hospital death. Patients were categorized into two groups according to the cutoff value: 524 with D-dimer ≤ 3.5 mg/L, 89 with D-dimer > 3.5 mg/L. Primary outcome occurred in 49.4% of patients with D-dimer > 3.5 mg/L, as compared with 26.3% in those with D-dimer ≤ 3.5 mg/L. The rate of in-hospital death (22.5% vs. 7.3%, *P* < 0.001, Fig. 1), embolic events (33.7% vs 18.2%, *P* = 0.001, Fig. 1) and stroke (29.2% vs 15.8%, *P* = 0.002, Fig. 1) was significantly higher in patients with D-dimer levels > 3.5 mg/L than in those without elevated D-dimer levels.

Univariable logistic regression analysis indicated that D-dimer was associated with increased in-hospital adverse events (OR = 1.14, *P* < 0.001) (Table 2). Additional significant indicators included RHD, CHD, prosthetic valve, NYHA III-IV heart failure, serum creatinine > 2 mg/dL, anemia, positive blood culture, perivalvular complications and surgical treatment. After adjusting the confounding variables, increased D-dimer was an independent risk factor for in-hospital adverse events (adjusted OR = 1.11, 95% CI = 1.03–1.19, *P* = 0.005, Table 2).

**Table 2:** Univariable and multivariable logistic regression analysis for in-hospital adverse events

Variables	Univariable			Multivariable		
	OR	95% CI	*P* value	OR	95%	*P* value
D-dimer	1.14	1.07, 1.21	< .001	1.11	1.03, 1.19	.005
Age	1.00	0.99, 1.01	.204			
Gender	1.02	0.70, 1.48	.910			
Diabetes mellitus	1.15	0.61, 2.20	.653			
Hypertension	1.44	0.91, 2.28	.115			
RHD	1.97	1.28, 3.02	.002	0.87	0.90, 3.27	.099
CHD	0.42	0.27, 0.64	.000	0.68	0.31, 1.46	.316
Prosthetic valve	2.15	1.20, 3.87	.010	3.69	1.44, 9.46	.007
NYHA III-IV	1.53	1.07, 2.20	.018	5.39	2.79, 10.42	< .001
SCr > 2.0 mg/dL	2.37	1.04, 5.39	.039	0.73	0.26, 2.06	.732
Anemia^a^	1.85	1.05, 3.26	.033	1.72	0.90, 3.27	.099
LVEF	0.98	0.96, 1.01	.311			
Vegetation > 10 mm	2.00	0.94, 4.26	.071			
Blood culture +	0.51	0.36, 0.73	< .001	0.64	0.34, 1.20	.160
PVC	1.85	1.09, 3.1	.022	2.09	0.90, 4.81	.085
No surgery	2.50	1.75, 3.57	< .001	5.67	2.85, 11.28	< .001

RHD, rheumatic heart disease; CHD, congenital heart disease, NYHA, New-York Heart Association; LVEF, left ventricular ejection fraction; PVC, perivalvular complications (refer to echocardiographic findings of perivalvular leak or abscess)

^a^Anemia is defined as hemoglobin < 110 g/L in male and < 110 g/L in female

### D-dimer and six-month mortality

**Fig. 2:**
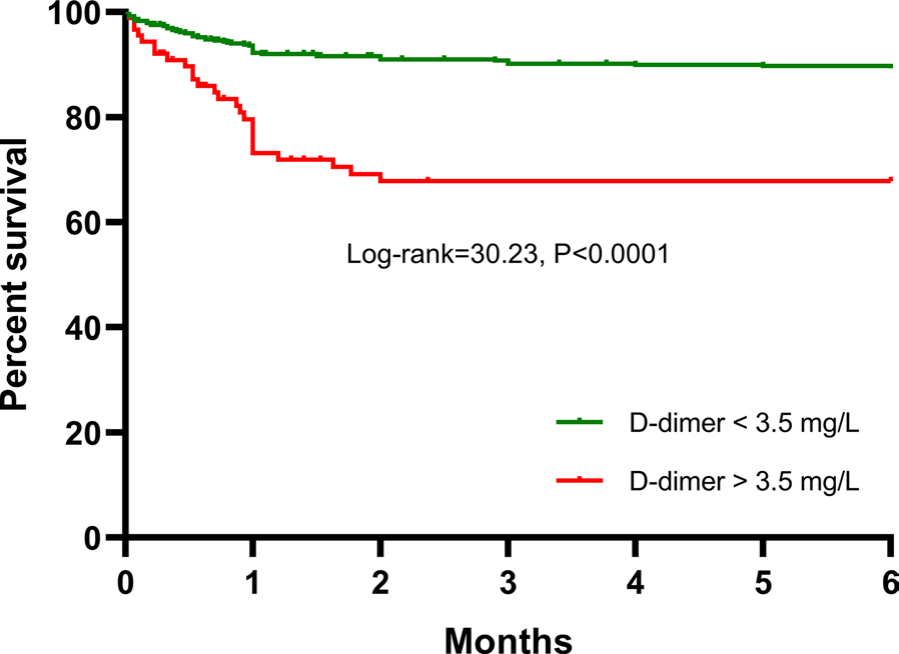
Kaplan–Meier survival curve of six-month survival in patients with IE stratified by the D-dimer cutoff

After six months, 80 patients died. Cumulative six-month mortality was significantly higher in the group with D-dimer > 3.5 mg/L than that in the other group (29.2% vs 10.3%, *P* < 0.001). Kaplan–Meier survival curve showed that patients with D-dimer levels > 3.5 mg/L had a lower six-month survival than those with D-dimer ≤ 3.5 mg/L (log-rank test = 30.23, *P* < 0.0001, Fig. 2). Multivariable Cox proportional hazard analysis showed that D-dimer remained a significant predictor for six-month death (HR 1.11, 95% CI 1.05–1.18, *P* < 0.001, Table 3).

**Table 3:** Multivariable Cox proportional hazard for six-month death

Clinical variables	Six-month death		
	HR	95% CI	*P* value
D-dimer	1.11	1.05, 1.18	< .001
RHD	1.60	0.80, 3.18	.183
CHD	0.79	0.39, 1.58	0.497
Prosthetic valve	2.68	1.27, 5.68	.010
NYHA III-IV	4.50	2.57, 7.88	< .001
SCr > 2 mg/dL	0.89	0.40, 1.95	.765
Vegetation > 10 mm	2.85	1.46, 5.55	.002
Blood culture +	0.55	0.33, 0.94	.027
PVC	1.40	0.66, 2.97	0.379
No surgery	7.84	4.15, 14.79	< 0.001

HR, hazard ratio; RHD, rheumatic heart disease; CHD, congenital heart disease, NYHA, New-York Heart Association; LVEF, left ventricular ejection fraction; PVC, perivalvular complications (refer to echocardiographic findings of perivalvular leak or abscess); SCr, serum creatinine

## Discussion

This study investigated the association of D-dimer at admission with the in-hospital adverse events and six-month mortality in a large cohort of patients with IE. Elevation of at-admission D-dimer level was associated with increased rate of in-hospital adverse events and six-month death. D-dimer was an independent predictor for both in-hospital adverse events and six-month mortality.

Early and accurate identification of patients at high risk and timely surgical intervention have been found to improve prognosis in patients with IE [14, 15]. However, the clinical history of IE is highly variable, rendering more focus on identifying predictors of adverse outcomes. Systemic embolization and in particular, CNS embolization, is one of the determinants of adverse outcome in patients with IE [2, 7, 16]. Therefore, early detection of activation of the coagulation cascade could play a pivotal role in recognizing excessive thrombus formation. Previous studies have proposed some clinical and laboratory predictors, including pro-inflammatory and hemodynamic biomarkers [17–20]. However, they are not directly related to thromboembolism. Identification of novel prognostic biomarkers on the basis of coagulation may further stratify patients with IE based on risk, providing guidance for clinical decision-making.

Elevated D-dimer level in plasma is indicative of acute thrombus formation and fibrinolysis, which is likely a valuable tool to diagnose a vast array of thrombosis-related clinical conditions [21]. Understanding the pathophysiology of IE might be helpful to completely elucidate the underlying mechanism of the prognostic role of D-dimer in IE. The characteristic endocardial lesion in IE—a vegetation—is an aggregation of platelets, fibrin, microorganisms, and inflammatory cells [2, 22]. Development of non-bacterial thrombotic endocarditis which is composed of a platelet–fibrin network is the nidus for bacterial adhesion and invasion. The bacteremia and colonization of bacteria on heart valves further promote platelet aggregation, coverage of the bacteria by a platelet–fibrin meshwork, and formation of mature vegetation [23]. This process involves recruitment of inflammatory cells, release of pro-inflammatory cytokines, and activation of the coagulation cascade. Pro-inflammatory cytokines and other mediators are capable of activating the coagulation system and down-regulating physiologic anticoagulant pathways and fibrinolysis, which reversely modulate the inflammatory process through protease-activated cell receptors and activation of platelets. Hence, down regulation of anticoagulant pathways not only promotes thrombosis but also amplifies the inflammatory process. This interplay of inflammation and coagulation, contributing significantly to the outcome, is one of the most prominent features of sepsis [24]. When the inflammation-coagulation interactions overwhelm the natural defense systems, catastrophic events such as those manifested in sepsis and IE occur. Furthermore, the continued proliferation of bacteria and deposition of platelets and fibrin result in vegetation that can embolize peripherally and cause embolic phenomena. As showed in our study, patients with elevated levels of on-admission D-dimer had a significantly higher rate of in-hospital thromboembolic events than those that did not. Embolism was the most common adverse events in our study cohort, attributing to 70% of all in-hospital events.

The prognosis of IE could be influenced by many factors. Previous studies have identified several predictors for in-hospital and long-term mortality in patients with IE, such as prosthetic heart valve, staphylococcus infection, LVEF, surgical therapy, vegetation size > 10 mm, perivalvular abscess and the presence of complications (stroke, heart failure, renal failure) [1, 5, 7, 12]. After adjusting these risk factors, D-dimer remains an independent predictor for in-hospital and 6-month all-cause mortality in our study. Besides, the presence of NYHA III-IV heart failure, negative blood culture and absence of surgical therapy were also identified as independent risk factors, which is consistent with previous studies. This finding supports the application of D-dimer in clinical practice to acquire additional prognostic information apart from traditional risk factors, especially when D-dimer > 3.5 mg/L.

Currently, many studies have been conducted to investigate the prognostic value of D-dimer in several thrombosis-related conditions. On-admission plasma D-dimer level has been reported to be a valuable marker in predicting short- and long-term outcomes in acute aortic dissection [25]. Similar results were found in patients with ST-elevation myocardial infarction undergoing primary percutaneous coronary intervention [26]. Musicant et al. reported that baseline D-dimer level was significantly associated with the occurrence of myocardial infarction in subjects with symptomatic peripheral artery disease [27]. To date, however, only four studies have been performed to evaluate the role of D-dimer in outcome prediction in IE. In a study including 42 patients, Bakal et al. showed that plasma D-dimer levels were increased in patients with IE who suffered from clinically significant systemic embolism; D-dimer level > 425 ng/dL could predict clinical embolism with a sensitivity of 77% and specificity of 62% [28]. However, the association of D-dimer level with in-hospital mortality in patients with IE was not studied. Turak et al. included 157 patients with IE and found that on-admission D-dimer ≥ 4.2 mg/L was independently associated with IE-related in-hospital death [12]. Recently, Baris et al. enrolled 79 patients with IE, and using multiple logistic regression analysis, showed that D-dimer was a strong parameter for predicting in-hospital mortality and embolic events [29]. Nevertheless, the small sample size and lack of long-term follow-up limit the significance of their results. Our study was conducted in a relatively large sample of patients, and the long-term follow-up demonstrated that increased D-dimer level on admission was independently associated with adverse in-hospital and long-term outcome in patients with IE. Additionally, this association was still significant after adjusting for cardiac function and surgical therapy, which have been shown as strong predictors for poor outcome in the previous studies and the current guideline. Therefore, we believe our study results provide further evidence for supporting D-dimer as a reliable biomarker to predict increased risk of complications and in-hospital and long-term death in patients with IE. Patients diagnosed with IE with increased D-dimer level should be closely monitored for embolization and carefully evaluated for early surgical intervention.

## Limitations

Our study has some limitations. First, it is a single center study and clinical data were retrospectively collected from electronic medical records. Our results should be interpreted with caution and require validation by prospective, multi-center studies. Second, reasons of death cannot be clearly identified by telephone follow-up. Third, Third, some peri-operative parameters that may be associated with patient outcome were not available in our study and not included into the analysis. Fourth, we only recorded on-admission D-dimer level without serial measurement. Measurement of D-dimer levels after treatment or upon discharge would be helpful to further analyze the correlation with disease progression and evaluate long-term outcome.

## Conclusion

Prognostic evaluation of patients with IE is of utmost clinical interest, which often guides clinicians to develop an algorithm for risk stratification and decision-making. Our study suggested that increased on-admission D-dimer was a reliable prognostic biomarker that associated with high risk of in-hospital adverse events and six-month mortality in patients with IE.

## Data Availability

The datasets used or analyzed during the current study are available from the corresponding author on reasonable request.

## Abbreviations

CRP: C-reactive protein
CHD: Congenital heart disease
CI: Confidence interval
DM: Diabetes mellitus
eGFR: Estimated glomerular filtration rate
HR: Hazard ratio
IE: Infective endocarditis
LVEF: Left ventricular ejection fraction
RHD: Rheumatic heart disease
ROC: Receiver operating characteristic
SCr: Serum creatinine
TEE: Transesophageal echocardiography
TTE: Transthoracic echocardiography
